# Fracture Healing and Commonly Co-Prescribed Medications: Orthopedic Implications in Multimorbid Patients: A Narrative Review

**DOI:** 10.7759/cureus.106315

**Published:** 2026-04-02

**Authors:** Yash Sharma, Babajide Obidigbo, Ikedichim Nwankwo

**Affiliations:** 1 Trauma and Orthopaedics, Royal Victoria Hospital, Belfast, Belfast, GBR; 2 Orthopaedics and Trauma, London Northwest University Healthcare NHS Trust, London, GBR; 3 Emergency, University of Port Harcourt, Port-Harcourt, NGA

**Keywords:** fracture healing, medication effects, orthopedic outcomes, polypharmacy, union outcomes

## Abstract

Fracture healing is a biologically complex process influenced not only by mechanical stability and patient-related factors but also by systemic medications commonly prescribed in orthopedic populations. In contemporary practice, many adults sustaining fractures are treated within a broader context of multimorbidity and polypharmacy, raising clinical questions about how commonly co-prescribed drug classes may intersect with biological pathways relevant to bone repair and recovery. However, evidence addressing these relationships remains dispersed across disciplines and is characterized by heterogeneity in study design, exposure definitions, and outcome measures.

To contextualize current evidence, we conducted a narrative review of peer-reviewed literature identified primarily through PubMed/MEDLINE, supplemented by manual screening of reference lists from key articles and reviews. The review focused on adult fracture populations and perioperative orthopedic settings and synthesized findings from observational studies, randomized trials, systematic reviews, and meta-analyses examining associations between fracture-related outcomes and medication classes frequently encountered in routine practice. These medication classes were evaluated individually rather than as interacting polypharmacy regimens, while remaining situated within the broader clinical context of multimorbidity and cumulative medication burden. These included analgesics and nonsteroidal anti-inflammatory drugs, systemic corticosteroids, anticoagulants, psychotropic medications, proton pump inhibitors, antidiabetic therapies, and other bone-active agents.

Across the literature, several medication classes have been associated with altered fracture-related or skeletal outcomes relevant to orthopedic recovery in specific clinical contexts, although findings were inconsistent and often highly context-dependent. Evidence linking nonsteroidal anti-inflammatory drugs, systemic corticosteroids, and anticoagulant therapies to fracture healing outcomes varied according to drug class, timing and duration of exposure, comorbidity burden, and fracture characteristics. For other commonly prescribed agents, including psychotropic medications, acid-suppressive therapies, and certain antidiabetic drugs, available data largely address fracture risk or bone health rather than fracture union or time to healing, limiting direct inference regarding repair processes. While some mechanisms underlying fracture risk, such as altered bone turnover or impaired bone quality, may overlap with pathways involved in repair, these processes are not biologically equivalent to the coordinated inflammatory, reparative, and remodeling phases of fracture healing and, therefore, require cautious interpretation.

Overall, current evidence suggests that fracture healing in adult orthopedic patients frequently occurs within a complex therapeutic landscape shaped by cumulative medication exposure and underlying metabolic and clinical factors. Rather than implicating polypharmacy as a single causal determinant of impaired union, these findings highlight the importance of individualized medication review and interdisciplinary care when managing fractures in patients with multimorbidity. Future research should prioritize prospective designs with standardized, fracture-healing-specific outcomes to better clarify medication-related risks and inform evidence-based orthopedic practice.

## Introduction and background

Fracture healing is a highly coordinated biological process that restores skeletal continuity through the interaction of inflammatory signaling, cellular recruitment, angiogenesis, and matrix remodeling. Experimental and clinical studies have demonstrated that bone repair progresses through overlapping inflammatory, reparative, and remodeling phases, each regulated at the molecular and cellular level [1,2]. Successful progression through these phases depends not only on local mechanical stability but also on systemic host conditions that influence cellular behavior and the tissue environment [3].

Conceptual frameworks of fracture repair emphasize that impaired healing is rarely attributable to a single factor. The so-called diamond concept highlights the combined importance of mechanical stability, osteogenic cells, growth factors, vascularity, and the host biological milieu in determining fracture union [4]. Disruption of one or more of these components may result in delayed union or nonunion, outcomes that continue to pose major clinical challenges despite advances in fixation techniques and perioperative care.

Nonunion and delayed healing impose a substantial burden on patients and healthcare systems. Established risk factors include fracture severity, soft-tissue injury, infection, smoking, and systemic conditions that alter inflammatory or metabolic pathways [5]. Large epidemiologic analyses show that nonunion rates vary considerably across anatomical sites and patient populations, with certain long bones and injury patterns associated with particularly high risk [6]. Population-level studies further demonstrate that delayed union, malunion, and nonunion are associated with prolonged disability, repeat surgical interventions, increased healthcare utilization, and higher direct and indirect costs following long-bone fractures [7].

In contemporary orthopedic practice, these biological and mechanical considerations increasingly intersect with complex systemic exposures. Aging populations and rising multimorbidity mean that many adults presenting with fractures are receiving long-term pharmacologic therapies for chronic conditions, in addition to medications prescribed during acute fracture care. Within this context, polypharmacy is common and reflects cumulative medication burden and care complexity rather than a single discrete or interaction-based exposure. Many commonly prescribed therapies have biological effects that plausibly intersect with pathways involved in fracture repair, including inflammation, vascular function, bone turnover, and cellular signaling. However, the extent to which individual medication classes influence fracture healing outcomes, as distinct from fracture risk or bone health more broadly, remains incompletely understood.

This review examines the orthopedic implications of systemic medications frequently encountered in adult fracture populations. It focuses on medication classes commonly co-prescribed in routine practice, including perioperative analgesics and nonsteroidal anti-inflammatory drugs, systemic corticosteroids and other immunomodulatory therapies, anticoagulants and antiplatelet agents, psychotropic medications, acid-suppressive therapies, antidiabetic treatments, and other bone-active agents. Given the heterogeneity of existing evidence, variation in exposure definitions, and inconsistent reporting of fracture healing endpoints, a narrative synthesis is well-suited to integrate findings across disciplines, clarify areas of consistency and uncertainty, and highlight clinically relevant knowledge gaps.

The objective of this narrative review is to synthesize peer-reviewed evidence on associations between commonly prescribed medications and fracture-related outcomes in adult orthopedic populations, situating these findings within the broader context of contemporary fracture management and polypharmacy. By framing medication effects within this clinical context, the review aims to support informed, individualized decision-making and to identify priorities for future research focused specifically on fracture healing outcomes.

Review design

This study was conducted as a narrative literature review examining how commonly prescribed systemic medications may relate to fracture healing and fracture-related outcomes in adult orthopedic populations. A narrative approach was selected to integrate evidence across heterogeneous study designs, medication exposures, and outcome definitions, which precluded formal quantitative pooling or systematic comparison. The review prioritizes clarity of scope, transparency of sources, and clinically meaningful organization rather than exhaustive coverage or systematic review methodology.

The reporting approach was informed by established guidance for narrative reviews, including the Scale for the Assessment of Narrative Review Articles (SANRA) and published recommendations on narrative synthesis and scholarly writing [8-10]. Preferred Reporting Items for Systematic Reviews and Meta-Analyses (PRISMA) guidelines were not applied, as the review was not designed to meet systematic review criteria. Nevertheless, key elements such as explicit eligibility considerations, transparent description of information sources, and structured organization were incorporated to enhance interpretability.

Information sources and search approach

Peer-reviewed literature was identified primarily through PubMed/MEDLINE. The most recent search was conducted in December 2025. Searches combined fracture-healing concepts with medication exposure terms and were refined iteratively to improve clinical relevance as recurring medication classes and outcome terminology emerged during screening.

A representative PubMed/MEDLINE search string was structured as follows: (“fracture healing” OR “fracture repair” OR “delayed union” OR “nonunion” OR “time to union”) AND (“nonsteroidal anti-inflammatory drugs” OR NSAIDs OR “COX-2 inhibitors” OR analgesics OR corticosteroids OR glucocorticoids OR anticoagulants OR warfarin OR “direct oral anticoagulants” OR antiplatelet OR “selective serotonin reuptake inhibitors” OR SSRIs OR antidepressants OR psychotropic OR “proton pump inhibitors” OR PPIs OR “acid suppression” OR diabetes OR “antidiabetic drugs” OR thiazolidinediones OR bisphosphonates OR statins).

Core fracture-healing terms and medication-related exposure categories were defined a priori and refined iteratively during screening. These included key fracture healing outcomes (e.g., delayed union, nonunion, and time to union) and commonly prescribed medication classes encountered in orthopedic populations, including analgesics and nonsteroidal anti-inflammatory drugs, corticosteroids, anticoagulants and antiplatelet agents, psychotropic medications, acid-suppressive therapies, antidiabetic agents, and selected bone-active therapies. Additional relevant studies were identified through manual screening of reference lists from high-yield systematic reviews, meta-analyses, and influential primary studies retrieved during the PubMed search.

Because this was a narrative review, the search and selection process was not intended to capture all eligible records for each medication class. Screening was oriented toward clinically influential syntheses and large, well-characterized clinical studies that were repeatedly cited across the fracture-healing literature. Priority was given to higher-level evidence, including systematic reviews and meta-analyses, as well as large prospective or well-characterized cohort studies, with consideration of study size, methodological clarity, and direct relevance to fracture-related outcomes.

Evidence selection was purposive rather than exhaustive. When multiple sources addressed the same question, priority was given to higher-level evidence (systematic reviews and meta-analyses) and large, well-characterized clinical cohort or case-control studies, supplemented by randomized trials where available. Mechanistic and translational studies were included selectively when they provided biologically plausible insights directly relevant to fracture repair or medication-related effects on bone biology. 

Table 1 summarizes the information sources, evidence types, and medication domains that structured the scope and organization of this narrative review.

**Table 1:** Search approach, evidence types, and scope of medication domains included in this narrative review NSAIDs: nonsteroidal anti-inflammatory drugs; SSRIs: selective serotonin reuptake inhibitors; PPIs: proton pump inhibitors. This narrative review was reported using established guidance for narrative review quality and synthesis [8-10].

Evidence domain	Primary information source(s) (as described in Methods)	Evidence types prioritized	Clinical focus in this review
Fracture healing biology and nonunion risk	PubMed/MEDLINE search; targeted manual reference screening of high-yield reviews and key primary studies	Narrative reviews, experimental/translational syntheses, epidemiologic studies	Biological phases of fracture repair; host and injury contributors to delayed union and nonunion
Analgesics and NSAIDs	PubMed/MEDLINE search; targeted manual reference screening	Systematic reviews/meta-analyses, cohort/case-control studies, randomized trials where available	Analgesic exposure in fracture care; associations with union-related outcomes (delayed union/nonunion/time to union)
Systemic corticosteroids and immunomodulatory therapy	PubMed/MEDLINE search; targeted manual reference screening	Narrative reviews, observational studies, mechanistic syntheses	Skeletal effects of systemic corticosteroids relevant to fracture repair context (indirect inference emphasized when union endpoints are limited)
Anticoagulants and antiplatelet therapy	PubMed/MEDLINE search; targeted manual reference screening	Population-based cohort/case-control studies, systematic reviews/meta-analyses	Skeletal outcomes across anticoagulant classes; union-specific evidence summarized when available, otherwise interpreted as indirect
Psychotropic medications (including SSRIs)	PubMed/MEDLINE search; targeted manual reference screening	Meta-analyses, population-based observational studies	Fracture risk and broader skeletal outcomes; limited direct evidence for union-specific endpoints
PPIs and acid suppression	PubMed/MEDLINE search; targeted manual reference screening	Observational studies, systematic reviews/meta-analyses	Associations between PPI exposure and fracture-related skeletal outcomes; healing-specific evidence noted when available
Metabolic disease and antidiabetic therapies	PubMed/MEDLINE search; targeted manual reference screening	Systematic reviews, mechanistic/clinical syntheses	Diabetes-related impairment of fracture outcomes; fracture risk signals for selected antidiabetic drug classes
Polypharmacy and prescribing context	PubMed/MEDLINE search; targeted manual reference screening	Reviews, consensus/position statements, deprescribing frameworks	Polypharmacy as a marker of multimorbidity and medication burden; implications for fracture care complexity and recovery environment
Other bone-active medications (adjunct exposures)	PubMed/MEDLINE search; targeted manual reference screening	Systematic reviews, clinical imaging/healing studies, selected experimental studies	Bisphosphonates and statins as examples of context-dependent skeletal effects; timing and clinical setting emphasized

Eligibility considerations

Included sources were peer-reviewed, English-language publications relevant to adult fracture populations and fracture-related outcomes. Eligible designs included randomized controlled trials, observational cohort and case-control studies, systematic reviews, and meta-analyses. Studies focused exclusively on pediatric populations, fracture incidence without relevance to healing or recovery, or outcomes unrelated to fracture repair were generally excluded. Editorials and narrative commentaries without primary data, and laboratory-only studies lacking clear clinical or translational relevance, were also excluded.

Data synthesis and organization

Evidence was synthesized narratively and organized by medication class, reflecting common patterns of prescribing and the manner in which exposures are encountered in fracture patients. Within each medication class, findings were summarized descriptively with attention to study design, population characteristics, exposure definitions, timing and duration of exposure, and reported outcomes. Where possible, fracture healing-specific endpoints (e.g., delayed union, nonunion, and time to union) were distinguished from broader skeletal outcomes such as fracture incidence or bone mineral density. When findings were inconsistent or context-dependent, these variations were described explicitly rather than reconciled into summary estimates. Interpretation emphasized clinical relevance and biological plausibility without implying causality.

Quantitative analysis and risk of bias

No formal risk-of-bias assessment tool was applied, and no quantitative pooling or meta-analysis was performed. This decision reflects the narrative design of the review and the heterogeneity of medication exposures, fracture types, and outcome definitions across the included literature. Quantitative findings reported by individual studies were considered as presented in the original sources and interpreted cautiously. While effect estimates were reviewed to inform interpretation, they were not systematically extracted or reported, given the heterogeneity in study designs, populations, and outcome definitions across the included literature. 

Ethics

This study synthesized published and publicly available literature and did not involve human participants, animal subjects, or identifiable data. Ethics approval was therefore not required.

This review does not evaluate polypharmacy quantitatively as an exposure; rather, the concept is used to contextualize cumulative medication burden and co-prescribing complexity in contemporary fracture care.

## Review

The sections presented below are used as organizational headings to structure the narrative synthesis by clinically relevant domains of medication exposure. They do not represent findings derived from formal qualitative analysis. 

Analgesics and nonsteroidal anti-inflammatory drugs

Nonsteroidal anti-inflammatory drugs (NSAIDs) feature prominently in the fracture healing literature because of their widespread use for musculoskeletal pain management and ongoing concern that they may influence union outcomes. A systematic review evaluating the quality of experimental and clinical evidence in this area highlighted substantial heterogeneity across studies, including variation in NSAID class, timing of exposure, fracture type, and outcome definitions, which limits generalizability across orthopedic settings [11].

Mechanistic concern has focused on cyclooxygenase inhibition and downstream effects on prostaglandin-mediated inflammatory signaling, which plays a recognized role in early fracture repair. Reviews of acute-phase healing emphasize that inflammation contributes to repair signaling, supporting the biological plausibility that NSAID exposure could influence callus formation or remodeling depending on timing and dose [12]. Accordingly, NSAIDs remain widely used for analgesia and opioid-sparing pain control in fracture patients, while continuing to be evaluated for potential associations with healing outcomes [12].

Clinical syntheses have produced mixed findings. Meta-analyses of randomized controlled trials suggest that short-term NSAID exposure may have limited or difficult-to-detect effects on healing, although trial designs and endpoints vary considerably [13]. In contrast, broader meta-analyses that incorporate observational studies more frequently report associations between NSAID exposure and delayed union or nonunion, though findings are sensitive to study selection, exposure definitions, and outcome ascertainment [14]. Critical appraisals consistently emphasize confounding by indication, fracture severity, and inconsistent outcome definitions as key limitations of observational analyses in this area [15]. In particular, patients with more severe or painful fractures may be more likely to receive prolonged or higher-dose NSAID therapy, which could bias observed associations toward an apparent increased risk of nonunion independent of a direct pharmacologic effect.

Pooled analyses of cohort and case-control studies have reported an increased risk of nonunion associated with NSAID exposure, with variable effect sizes and persistent potential for residual confounding [16]. Population-based analyses further suggest that associations may differ across analgesic classes, including distinctions between nonselective NSAIDs, selective cyclooxygenase-2 inhibitors, and opioid comparators, highlighting that class effects should not be assumed to be uniform [17]. In specific orthopedic contexts, indomethacin used for heterotopic ossification prophylaxis has been associated with increased nonunion risk in long-bone fractures [18].

Taken together, the literature supports a context-dependent interpretation rather than a uniform effect of NSAIDs on fracture healing. Where risk signals are observed, they appear most consistently linked to longer duration exposure, higher cumulative dose, early-phase use, and fracture or patient profiles already predisposed to delayed union or nonunion. Conversely, short-term analgesic use shows less consistent associations across studies and settings, underscoring the importance of individualized risk assessment when NSAIDs are considered during fracture care [11,14,15].

Corticosteroids and immunomodulatory therapy

Systemic corticosteroids are widely used for inflammatory, autoimmune, and respiratory conditions and are therefore encountered in a subset of patients presenting with fractures. Their relevance to fracture healing has been discussed predominantly in relation to glucocorticoid-induced effects on bone biology rather than through direct evaluation of fracture union outcomes. Most available evidence pertains to fracture incidence and bone mineral density rather than union or time-to-union; therefore, implications for fracture repair are indirect. Foundational reviews describe how chronic glucocorticoid exposure alters bone remodeling through suppression of osteoblast function, promotion of osteoblast and osteocyte apoptosis, and prolonged osteoclast survival, collectively contributing to reduced bone formation and impaired skeletal integrity [19].

Clinical literature has consistently linked long-term systemic corticosteroid use with decreased bone mineral density and increased fracture risk, establishing glucocorticoids as a major secondary cause of osteoporosis [19]. Subsequent syntheses emphasize that both dose and duration of exposure are critical determinants of skeletal harm, with higher cumulative doses conferring greater risk [20]. These systemic effects provide biological plausibility for impaired fracture repair in exposed patients; however, clinical studies directly evaluating fracture healing endpoints such as union, delayed union, or time to healing remain limited.

Epidemiologic analyses further contextualize corticosteroid-associated skeletal vulnerability by demonstrating that adverse effects on bone may occur early after treatment initiation and persist with ongoing exposure [21]. While most available data address fracture incidence rather than fracture healing per se, the well-established influence of glucocorticoids on bone turnover and repair-related pathways suggests that chronic exposure may represent a background modifier of fracture healing trajectories in orthopedic populations.

Overall, the evidence supports cautious interpretation rather than definitive conclusions. Corticosteroid exposure is best considered a contextual risk factor that may influence fracture healing indirectly through systemic effects on bone biology, with clinical relevance likely dependent on dose, duration of therapy, and the presence of additional risk factors for impaired union [19-21].

Anticoagulants, antiplatelet therapy, and vitamin K-dependent pathways

Oral anticoagulants are frequently prescribed in older orthopedic patients for atrial fibrillation, venous thromboembolism, and cardiovascular disease, and therefore represent a common systemic exposure in fracture populations. Most available evidence for this medication class pertains to fracture incidence or general skeletal outcomes rather than fracture union or time to healing; therefore, implications for fracture repair should be interpreted as indirect. Interest in their skeletal effects has focused largely on vitamin K antagonism, given the role of vitamin K-dependent proteins in bone mineralization and matrix regulation. Early epidemiologic studies reported an increased risk of osteoporotic fractures among elderly patients receiving long-term warfarin therapy, raising concern about potential downstream effects on skeletal health [22].

Subsequent cohort studies using propensity-matched designs supported an association between prolonged warfarin exposure and increased fracture risk, although these analyses primarily evaluated fracture incidence rather than fracture healing outcomes [23]. Comparative population-based studies further demonstrated that fracture risk varies across anticoagulant classes, with lower rates observed among users of direct oral anticoagulants compared with warfarin, highlighting the relevance of drug mechanism and class-specific effects [24]. National case-control analyses similarly identified higher fracture risk among users of oral anticoagulants, particularly vitamin K antagonists, while emphasizing the influence of age, comorbidity burden, and baseline fracture risk as important confounders [25]. More recent systematic reviews and network meta-analyses comparing direct oral anticoagulants with warfarin have consistently reported neutral or reduced fracture risk with newer agents, suggesting a more favorable skeletal safety profile relative to vitamin K antagonists [26].

Evidence directly examining fracture healing outcomes rather than fracture incidence remains limited. Clinical studies reporting union, delayed union, or time-to-union endpoints specifically in anticoagulant-exposed fracture cohorts remain uncommon [27]. Narrative syntheses addressing anticoagulant pharmacotherapy and fracture repair have described plausible mechanisms by which anticoagulants could influence healing processes, including effects on bone turnover and local hematoma formation, while acknowledging the scarcity of clinical data evaluating fracture union, delayed union, or time to healing [27]. Meta-analyses incorporating fracture outcomes across anticoagulant classes further indicate that skeletal effects are not uniform and may depend on drug mechanism and duration of exposure, though healing-specific endpoints are rarely reported [28].

Overall, current evidence suggests that anticoagulant therapy, particularly vitamin K antagonism, is associated with differences in skeletal outcomes at the population level. However, direct clinical evidence linking anticoagulant exposure to impaired fracture healing or fracture union remains sparse. As such, anticoagulant use is best interpreted as a contextual factor within fracture care rather than a proven determinant of healing outcomes, underscoring the need for cautious interpretation and individualized risk assessment in orthopedic practice.

Selective serotonin reuptake inhibitors and psychotropic medications

Psychotropic medications, particularly selective serotonin reuptake inhibitors (SSRIs), are commonly prescribed for depression, anxiety, and related conditions and are therefore frequently encountered in older adults and patients with multimorbidity presenting with fractures. Most available evidence for this medication class pertains to fracture incidence or general skeletal outcomes rather than fracture union or time to healing; therefore, implications for fracture repair should be interpreted as indirect. Interest in their skeletal effects has arisen primarily from observational studies reporting associations between SSRI exposure and increased fracture risk, prompting further investigation into potential effects on bone biology and orthopedic outcomes.

Meta-analyses of cohort and case-control studies have consistently reported an association between SSRI use and increased fracture incidence across diverse populations [29,30]. These analyses suggest that risk may be influenced by duration of exposure and cumulative dose, although substantial heterogeneity exists across studies. Subsequent meta-analyses incorporating broader classes of antidepressants have reinforced these findings while noting that fracture risk varies by medication class and patient characteristics [31].

Population-based studies examining psychotropic medications more broadly have demonstrated increased fracture risk associated with antidepressants and other centrally acting agents, particularly in older adults in whom fall risk, frailty, and baseline skeletal vulnerability often coexist [32]. Large cohort analyses have similarly reported higher fracture incidence among SSRI users compared with nonusers, even after adjustment for measured confounders, although residual confounding related to underlying psychiatric illness and functional status cannot be fully excluded [33].

Direct evidence linking SSRI exposure to fracture healing outcomes, as distinct from fracture incidence, remains limited. Most available studies focus on fracture occurrence rather than endpoints such as delayed union, nonunion, or time to healing, and few explicitly evaluate post-fracture repair trajectories. Proposed mechanisms include serotonergic effects on osteoblast and osteoclast activity, as well as indirect influences mediated through fall risk, comorbidity burden, and functional impairment, which together provide biological plausibility for potential effects on fracture recovery without establishing a causal relationship [29-31].

Overall, the literature supports a consistent association between SSRI use and increased fracture risk, while evidence directly addressing fracture healing outcomes is sparse. In orthopedic populations, SSRI exposure is therefore best interpreted as a contextual factor influencing fracture risk and recovery environment rather than as a proven determinant of impaired fracture union.

Proton pump inhibitors and acid suppression

Proton pump inhibitors (PPIs) are widely prescribed for gastroesophageal reflux disease, peptic ulcer disease, and gastrointestinal protection in patients receiving nonsteroidal anti-inflammatory drugs. As a result, long-term PPI use is common among older adults and patients with multimorbidity, who also constitute a substantial proportion of orthopedic fracture populations. Most available evidence for this medication class pertains to fracture incidence or general skeletal outcomes rather than fracture union or time to healing; therefore, implications for fracture repair should be interpreted as indirect. Interest in potential skeletal effects has arisen primarily from epidemiologic studies reporting associations between acid suppression and fracture risk.

Early large observational studies reported an association between prolonged PPI therapy and increased hip fracture risk, prompting concern regarding possible adverse skeletal effects with long-term use [34]. Subsequent meta-analyses pooling international observational data supported a modest association between PPI exposure and fracture incidence, with pooled relative risk estimates indicating small but statistically significant increases that varied by fracture site, although heterogeneity across studies was substantial [35]. These findings stimulated further investigation into potential mechanisms linking acid suppression to bone health.

However, longitudinal studies examining bone mineral density have not consistently demonstrated accelerated bone loss among PPI users, suggesting that observed associations with fracture risk may not be mediated through clinically meaningful reductions in bone density [36]. Systematic reviews and meta-analyses have emphasized the inconsistency of available findings and highlighted the likelihood of residual confounding related to age, comorbidity burden, frailty, and concurrent medication use [37].

Evidence directly evaluating fracture healing or fracture union outcomes in the context of PPI exposure remains limited. Most available studies address fracture incidence rather than post-fracture repair, and mechanistic pathways linking acid suppression to impaired fracture healing have not been clearly established. Taken together, current evidence does not support a clear or consistent role for PPIs as modifiers of fracture healing trajectories. In orthopedic practice, PPIs are therefore best regarded as common background exposures rather than modifiable drivers of impaired fracture union.

Metabolic disease, polypharmacy, and cross-cutting bone-active medications

Metabolic disease and cumulative medication exposure provide an important clinical backdrop for fracture healing in contemporary orthopedic practice. Diabetes mellitus, in particular, has been associated with poorer fracture-related outcomes in adult populations, including signals consistent with delayed healing and higher complication rates in some settings [38]. Alongside disease-related effects, medication exposures common in diabetes management may influence skeletal vulnerability. A meta-analysis reported increased fracture risk with long-term thiazolidinedione use in type 2 diabetes, reinforcing that treatment choices can contribute to skeletal vulnerability, although these data do not directly evaluate union or time-to-union outcomes [39]. Mechanistic and clinical syntheses further describe how hyperglycemia, microvascular dysfunction, and altered inflammatory signaling impair bone quality and cellular processes involved in fracture repair, providing biological plausibility for delayed recovery in diabetes [40].

Beyond single disease states, the concurrent use of multiple medications is increasingly prevalent in the age groups most affected by fragility fractures and reflects multimorbidity, cumulative treatment burden, and care complexity rather than a discrete pharmacologic exposure. Reviews of polypharmacy in older adults emphasize associations with adverse clinical outcomes such as falls, drug interactions, and physiological stress, all of which may complicate postoperative recovery and rehabilitation following fracture [41,42]. Broader prescribing frameworks highlight that optimizing medication regimens in patients with multimorbidity requires balancing competing therapeutic priorities and minimizing potentially inappropriate prescribing, a challenge that is particularly relevant during transitions between orthopedic, medical, and rehabilitation care settings [43]. Consensus recommendations and deprescribing guidance similarly stress the role of structured medication review in reducing avoidable harm while maintaining essential therapies [44,45].

Several bone-active medications commonly encountered in fracture populations illustrate why medication effects are best interpreted as context-dependent rather than uniformly detrimental to healing. Bisphosphonates, for example, alter bone remodeling dynamics, and systematic review evidence suggests that their influence on fracture healing is not consistently adverse across clinical contexts [46]. Experimental studies have also suggested anabolic effects of statins on bone formation, although translation to consistent clinical benefit in fracture repair remains uncertain [47]. In orthopedic populations, radiographic healing studies evaluating bisphosphonate exposure further highlight the importance of timing, fracture site, and patient selection when interpreting potential effects on fracture union [48].

More broadly, orthopedic syntheses of impaired fracture healing emphasize that delayed union and nonunion typically arise from multiple converging factors rather than from a single medication exposure. These factors include biological vulnerability, metabolic disease, comorbidity burden, and local or mechanical considerations related to injury and fixation [49]. Systematic review evidence examining statins and fracture outcomes similarly reports mixed findings across populations and study designs, reinforcing that medication-associated skeletal effects are heterogeneous and frequently confounded by underlying patient risk [50].

Taken together, these findings indicate that fracture healing commonly occurs within a complex therapeutic landscape shaped by metabolic disease and cumulative medication exposure. The clinical implication is not that commonly prescribed medications should be routinely discontinued but that medication review should be individualized and integrated with fracture risk assessment, comorbidity management, and rehabilitation planning as part of comprehensive fracture care [41,45].

Table 2 provides a concise summary of the major medication classes discussed, the nature of available evidence, and their contextual implications for fracture-related outcomes.

**Table 2:** Summary of commonly prescribed medication classes and their contextual implications for fracture-related outcomes NSAIDs: nonsteroidal anti-inflammatory drugs; SSRIs: selective serotonin reuptake inhibitors; PPIs: proton pump inhibitors.

Medication class	Typical clinical context in fracture patients	Biological considerations relevant to fracture repair	Nature of available evidence	Overall interpretation
Nonsteroidal anti-inflammatory drugs (NSAIDs)	Acute analgesia and opioid-sparing pain control	Cyclooxygenase inhibition may alter prostaglandin-mediated inflammatory signaling involved in early fracture repair	Experimental studies, observational cohorts, randomized trials, and meta-analyses	Mixed and context dependent; potential risk signals with prolonged or early high-dose exposure, but no uniform effect on healing [11–16]
Systemic corticosteroids	Chronic inflammatory, autoimmune, or respiratory disease	Suppression of osteoblast activity, increased osteocyte apoptosis, and altered bone remodeling	Observational studies and mechanistic reviews	Indirect risk modifier; skeletal effects largely related to chronic exposure rather than acute fracture care [19–21]
Anticoagulants and antiplatelet therapy	Atrial fibrillation, venous thromboembolism, cardiovascular disease	Vitamin K antagonism may influence bone mineralization; direct oral anticoagulants differ mechanistically	Cohort studies, case-control analyses, systematic reviews	Class-specific effects on fracture risk; direct evidence on fracture healing remains limited [22–27]
Psychotropic medications (SSRIs and related agents)	Depression, anxiety, and other psychiatric conditions	Possible serotonergic effects on bone cells; indirect effects via falls, frailty, and comorbidity	Meta-analyses and population-based studies	Consistent association with fracture risk, but limited data addressing fracture union or time to healing [29–31]
Proton pump inhibitors (PPIs)	Gastroprotection, reflux disease, NSAID co-therapy	Hypothesized effects on mineral absorption; mechanisms remain uncertain	Observational studies and meta-analyses	Inconsistent evidence; available data do not support a clear role in impaired fracture healing [34–37]
Antidiabetic therapies and metabolic disease	Diabetes mellitus with or without insulin resistance	Hyperglycemia, microvascular dysfunction, and chronic inflammation may impair repair processes	Systematic reviews and mechanistic syntheses	Background biological risk; disease-related effects likely outweigh medication-specific effects [38–40]
Bisphosphonates	Osteoporosis management and secondary fracture prevention	Altered bone remodeling dynamics	Systematic reviews and clinical imaging/healing studies	Timing-dependent effects; generally not associated with impaired fracture union in clinical studies [46,48]
Statins	Dyslipidemia and cardiovascular disease	Experimental anabolic effects on bone formation	Experimental studies and clinical meta-analyses	Biological plausibility without consistent clinical benefit for fracture healing [47,50]
Polypharmacy (cumulative exposure)	Older adults with multimorbidity	Drug interactions, physiological burden, and indirect effects on recovery capacity	Geriatric and prescribing literature	Contextual risk factor, reflecting multimorbidity and care complexity rather than a discrete causal exposure [41–45]

Discussion

This narrative review examined how commonly prescribed medications intersect with fracture healing in adult orthopedic populations within the context of contemporary multimorbidity and co-prescribing. Rather than identifying a single pharmacologic determinant of impaired union, the literature collectively indicates that medication-related effects on fracture repair are variable and influenced by timing of exposure, duration of use, fracture characteristics, and patient comorbidity. Across medication classes, evidence is heterogeneous, underscoring the importance of contextualized interpretation rather than uniform clinical assumptions. Much of the available literature evaluates fracture risk or skeletal health rather than fracture healing outcomes. While factors such as reduced bone mineral density may increase susceptibility to fracture, they are not biologically equivalent to the temporally regulated inflammatory, reparative, and remodeling processes that govern fracture union and therefore require cautious interpretation when extrapolating to healing outcomes.

Nonsteroidal anti-inflammatory drugs have received the greatest attention in relation to fracture healing. Experimental and translational studies demonstrate that cyclooxygenase inhibition can modify prostaglandin-mediated inflammatory signaling, a process involved in early callus formation [12]. Clinical evidence, however, is less consistent. Meta-analyses incorporating randomized and observational data report mixed associations between NSAID exposure and delayed union, with effect estimates varying by study design and exposure duration [13,14]. Observational analyses suggest that prolonged or early postoperative NSAID use may be associated with higher nonunion risk in certain fracture types, whereas short-term or intermittent use shows weaker or inconsistent associations [16,17]. Taken together, these findings suggest that any potential NSAID-related risk, when observed, is most relevant in settings involving higher cumulative exposure, early-phase use, or fractures already predisposed to nonunion. Clinically, this supports judicious, time-limited use and individualized analgesic strategies rather than routine avoidance.

For corticosteroids, the literature more clearly supports adverse skeletal effects with chronic exposure, including reduced bone formation and increased fracture risk [19,21]. Reviews of glucocorticoid-induced osteoporosis highlight impaired osteoblast function and altered bone remodeling as central mechanisms [19,20]. However, direct evidence evaluating the impact of short-term or peri-fracture corticosteroid exposure on fracture healing remains limited, and most available data relate to long-term use rather than acute prescribing [21]. This distinction is important when extrapolating fracture risk data to fracture repair outcomes, particularly in patients requiring corticosteroids for acute medical indications.

Anticoagulant therapy introduces additional complexity. Population-based studies demonstrate increased fracture risk among users of vitamin K antagonists, likely related to interference with vitamin K-dependent bone pathways [22,23]. Comparative analyses indicate that direct oral anticoagulants may be associated with lower fracture risk relative to warfarin [24,26]. Reviews focusing on fracture healing emphasize that available evidence remains sparse and largely indirect, with most studies addressing fracture incidence rather than union or time to healing [27]. Consequently, while anticoagulant choice may influence overall skeletal risk profiles, its direct impact on fracture repair remains insufficiently characterized and should be interpreted cautiously in orthopedic decision-making.

In addition to class-specific effects, concurrent use of multiple medications may introduce clinically relevant influences that are not fully captured when drug classes are considered in isolation. For example, combinations that increase bleeding risk could plausibly affect the early fracture environment, including hematoma formation, which plays a role in the initial phase of repair [1,2,27]. More broadly, overlapping medication effects on inflammatory and repair-related pathways may modify the host biological milieu in ways that are difficult to disentangle in multimorbid patients [11,12,27]. However, clinical evidence directly evaluating such interaction-level effects on fracture union remains limited, and these considerations are best interpreted as biologically plausible rather than established determinants of healing outcomes [27].

Psychotropic medications, particularly selective serotonin reuptake inhibitors, have been consistently associated with increased fracture risk across multiple meta-analyses and population-based studies [29-31]. Proposed mechanisms include serotonergic effects on bone cells and indirect contributions from falls, frailty, and comorbid illness [32,33]. However, the literature rarely distinguishes fracture healing outcomes from fracture occurrence, limiting conclusions regarding direct effects on repair processes. Similar limitations apply to proton pump inhibitors, where observational studies and meta-analyses report modest associations with fracture risk, but mechanistic explanations remain uncertain, and evidence specific to fracture healing is lacking [34-36]. In both cases, associations with fracture risk should not be assumed to translate into impaired fracture union.

Metabolic disease further shapes the fracture healing environment. Systematic reviews and mechanistic syntheses demonstrate that diabetes mellitus is associated with delayed healing, increased complication rates, and poorer fracture outcomes, mediated by hyperglycemia, microvascular impairment, and chronic inflammation [38,40]. In this context, medication effects are often secondary to the underlying disease process. Among older adults, cumulative medication burden reflects multimorbidity and care complexity, contributing to risks such as drug interactions, physiological stress, and barriers to rehabilitation rather than acting as a single causal factor influencing fracture repair [41,43,45].

Taken together, these findings indicate that fracture healing commonly occurs within a complex therapeutic and metabolic landscape. The practical implication is not that commonly prescribed medications should be routinely discontinued but that medication review should be individualized and integrated with fracture risk assessment, comorbidity management, and rehabilitation planning. This approach aligns with contemporary principles of geriatric and orthopedic care, which emphasize balanced prescribing and interdisciplinary coordination over reflexive medication avoidance [41,45].

Practical considerations for clinicians

From a clinical perspective, the findings of this review support a measured and individualized approach to medication management in fracture patients rather than routine discontinuation of established therapies. Structured medication reconciliation at key transition points, including emergency department presentation, perioperative planning, and hospital discharge, provides an opportunity to identify potentially modifiable risks while maintaining necessary treatment for comorbid conditions.

For analgesia, nonsteroidal anti-inflammatory drugs may be used judiciously, with particular caution regarding prolonged duration, higher cumulative doses, and early-phase exposure in fractures already at elevated risk for delayed union or nonunion. In such contexts, shared decision-making, time-limited use, and consideration of alternative or adjunctive pain control strategies may help balance analgesic benefit against potential healing concerns.

For anticoagulant therapy, current evidence does not support medication changes solely to optimize fracture healing. Where multiple therapeutic options are clinically appropriate, consideration of overall skeletal risk, comorbidity burden, and bleeding risk may inform individualized decision-making. Importantly, fracture incidence data should not be extrapolated directly to fracture healing outcomes in the absence of union-specific evidence.

For psychotropic medications and acid-suppressive therapies, available data largely relate to fracture risk rather than fracture repair. Clinical focus may therefore be better directed toward fall risk mitigation, nutritional status, and optimization of bone health, where appropriate, rather than medication withdrawal based on indirect evidence.

Research priorities

Future research should prioritize prospective study designs that explicitly evaluate fracture healing outcomes in relation to medication exposure, with predefined exposure windows, standardized definitions of union, and clinically relevant follow-up intervals. Clear separation of fracture incidence outcomes from fracture healing endpoints is essential to improve interpretability and clinical relevance.

Greater attention to cumulative medication burden and drug interaction profiles, rather than isolated single-drug effects, may better reflect real-world fracture populations characterized by multimorbidity and co-prescribing. Future studies may benefit from advanced analytical approaches, including interaction-term modeling, propensity-based methods, and the use of large clinical or orthogeriatric registries, to better characterize cumulative exposures and interaction-level effects on fracture healing outcomes. Integrating pharmacologic exposure data with fracture characteristics, metabolic status, and rehabilitation factors may help clarify how systemic therapies interact with the biological processes of fracture repair.

Strengths and limitations

This review integrates evidence across multiple medication classes relevant to real-world fracture care, drawing on experimental, observational, and synthesis studies. The narrative approach allowed consideration of biological plausibility alongside clinical data, which is particularly important where direct fracture healing evidence is limited. However, several limitations warrant consideration. The narrative design does not permit quantitative pooling or formal risk-of-bias assessment, and heterogeneity in outcome definitions limits comparability across studies. For many medication classes, direct evidence evaluating fracture healing outcomes is limited, with much of the available literature focusing on fracture risk rather than union or time to healing.

Additionally, heterogeneity in fracture type, treatment approach, medication timing, and exposure duration limits cross-study comparability. The literature is also subject to confounding by indication, particularly in observational studies where underlying disease severity may influence both medication use and fracture outcomes. Finally, as this review did not apply a reproducible systematic search strategy, it cannot be considered exhaustive, although efforts were made to include clinically influential and higher-level evidence relevant to contemporary orthopedic practice.

## Conclusions

Fracture healing occurs within a complex clinical context shaped by injury characteristics, comorbidity burden, and cumulative medication exposure. The available evidence does not support uniform assumptions regarding the impact of commonly prescribed medications on fracture repair. Instead, reported effects are agent-specific, timing-dependent, and closely intertwined with underlying metabolic, biological, and clinical factors. Across medication classes, much of the literature addresses fracture risk or skeletal health rather than fracture healing outcomes, underscoring the need for cautious interpretation when extrapolating findings to union or recovery trajectories.

For clinicians, these findings reinforce the importance of individualized medication review rather than routine discontinuation of established therapies during fracture care. Integration of orthopedic, pharmacologic, and medical perspectives is essential to balancing analgesia, thromboprophylaxis, and chronic disease management while supporting fracture recovery in patients with multimorbidity. Future research should prioritize prospective study designs with standardized, fracture-healing-specific endpoints and clearly defined exposure windows to better inform evidence-based medication management in orthopedic practice.
